# Insightful Valorization of the Biological Activities of Pani Heloch Leaves through Experimental and Computer-Aided Mechanisms

**DOI:** 10.3390/molecules25215153

**Published:** 2020-11-05

**Authors:** Naureen Banu, Najmul Alam, Mohammad Nazmul Islam, Sanjida Islam, Shahenur Alam Sakib, Nujhat Binte Hanif, Md. Riad Chowdhury, Abu Montakim Tareq, Kamrul Hasan Chowdhury, Shamima Jahan, Afrina Azad, Talha Bin Emran, Jesus Simal-Gandara

**Affiliations:** 1 Department of Pharmacy, International Islamic University Chittagong, Chittagong 4318, Bangladesh; naureen2021@gmail.com (N.B.); nazmul9alam@gmail.com (N.A.); sanjida.ru@outlook.com (S.I.); sakibhasaniiuc@gmail.com (S.A.S.); nujhataanchol@gmail.com (N.B.H.); riadchy01@gmail.com (M.R.C.); montakim0.abu@gmail.com (A.M.T.); kamrulhasan73132@gmail.com (K.H.C.); shamimaj058@gmail.com (S.J.); 2 Department of Theoretical and Computational Chemistry, University of Dhaka, Dhaka 1000, Bangladesh; 3 Bangladesh Council of Scientific and Industrial Research (BCSIR), Dr. Qudrat-i-Khuda Road, Dhanmondi, Dhaka 1205, Bangladesh; afrina.ru.pharm@gmail.com; 4 Department of Pharmacy, BGC Trust University Bangladesh, Chittagong 4381, Bangladesh; 5 Nutrition and Bromatology Group, Department of Analytical and Food Chemistry, Faculty of Food Science and Technology, University of Vigo—Ourense Campus, E32004 Ourense, Spain

**Keywords:** *Antidesma montanum*, Pani heloch, antioxidant, anti-inflammatory, thrombolytic, analgesic, antipyretic, ADME/T

## Abstract

Pani heloch (*Antidesma montanum*) is traditionally used to treat innumerable diseases and is a source of wild vegetables for the management of different pathological conditions. The present study explored the qualitative phytochemicals; quantitative phenol and flavonoid contents; in vitro antioxidant, anti-inflammatory, and thrombolytic effects; and in vivo antipyretic and analgesic properties of the methanol extract of *A. montanum* leaves in different experimental models. The extract exhibited secondary metabolites including alkaloids, flavonoids, flavanols, phytosterols, cholesterols, phenols, terpenoids, glycosides, fixed oils, emodines, coumarins, resins, and tannins. Besides, Pani heloch showed strong antioxidant activity (IC_50_ = 99.00 µg/mL), while a moderate percentage of clot lysis (31.56%) in human blood and significant anti-inflammatory activity (*p* < 0.001) was achieved with the standard. Moreover, the analgesic and antipyretic properties appeared to trigger a significant response (*p* < 0.001) relative to in the control group. Besides, an in silico study of carpusin revealed favorable protein-binding affinities. Furthermore, the absorption, distribution, metabolism, excretion, and toxicity analysis and toxicological properties of all isolated compounds adopted Lipinski’s rule of five for drug-like potential and level of toxicity. Our research unveiled that the methanol extract of *A. montanum* leaves exhibited secondary metabolites that are a good source for managing inflammation, pyrexia, pain, and cellular toxicity. Computational approaches and further studies are required to identify the possible mechanism which responsible for the biological effects.

## 1. Introduction

Natural medicinal plants are extensively used across the world in the prevention of chronic diseases, including mild to moderate and severe illnesses [1]. Despite emerging technology and advancements in modern medicine, a large portion of the world’s population still depends upon traditional medicine [2]. Public interest in herbs is growing exponentially day by day [3]. Living cells (i.e., DNA, RNA, protein, and lipids) are impaired due to the overproduction of free radicals, which play an important role in the generation of various chronic diseases such as atherosclerosis, cardiovascular diseases, respiratory disorders, inflammation, rheumatoid arthritis, ischemic stroke, cancer, and other neurodegenerative diseases [4]. Hence, antioxidants play a vital role in either scavenging or decreasing the development of free radicals so as to support recovery from numerous diseases [5].

Many diseases and injuries are marked by the onset of pain and fever [6], yet pain, pyrexia, and inflammation can also develop individually with different physiological responses [7]. Inflammation is a complex natural reaction of the vascular tissue in response to increased sensitivity and the mediator’s attempt to protect and evacuate harmful stimuli [8]. The pyrexia is a powerful biological response modifier [9] that regulates the mind and internal level of heat. The nerve center transmits signals depending upon the arrival of pyrogens in the body, which constitute a class of biochemical substances discharged for tissue scarring or microbial contamination [10]. Nonsteroidal anti-inflammatory drugs (NSAIDs) are widely used but severe gastrointestinal complications such as ruptured bowel, peptic ulcers, and bleeding have limited their application in clinical conditions [11,12]. Cyclooxygenase-2 (COX-2) might have some advantages in minimizing certain side effects, but its use requires special considerations in cardiovascular problems [13,14,15]. At least eight different kinds of opioid receptors have been identified to date, yet the respective functions have only begun to be understood [7]. In addition, lipid mediators have great implications in the inflammatory response, while salicylates and other NSAIDs are effective in regulating the prostaglandin-induced release of pyrogens [7].

Presently available pain-relieving medications are not so successful at reducing pain or, in some cases, only contribute to a 50% reduction in the pain level at most, suggesting a desperate need for more effective analgesics in approximately 30% of patients [16]. Moreover, the discovery and development of novel medications to relieve pain and fever are important to reduce the side effects and problems associated with current analgesics [17,18]. Natural sources are important options for alternative medicines in pain and fever, whereas almost 25% of synthetic drugs are directly or indirectly derived from medicinal plants [17]. Medicinal plants are suitable sources for the development of new drugs as they are typically plentiful with long use histories and limited side effects. The purpose of our current study was to assess the biological response to Pani heloch (*Antidesma montanum*) with computational analysis to minimize inflammation, pyrexia, pain, and cell toxicity.

*Antidesma montanum* Blume (Pani heloch) is a member of the family Euphorbiaceae, under the subfamily Phyllanthaceae, and the leaves of *A. montanum* have been considered as a wild vegetable [19,20], while traditional physicians have adopted different parts of the plant to treat ulcers, eye disease, lumber pain, hematochezia [21]. It is the most familiar and widespread of all species, and the plant is colloquially referred to as “Pani heloch.” The roots have been used internally to cure malaria, chickenpox, and measles and the leaf has been externally applied for thrush in children and headache in Malaysia [19]. The plant has also been used by native people in Indonesia for the healing of diabetes and is used as a medicinal plant in the Limu Mountains of China for the management of eye diseases [22]. The root was also used to prepare tea and the leaves have been reported as a means to relieve chest pain, dermatitis, and other skin diseases [23]. In previous research, the methanol extract presented some major isolated compounds of this plant—notably, 3,7,11,15-tetramethyl-2-hexadecen-1-ol, 9-ecosyne, hexadecanoic acid, gamma sitosterol, tridecanoic acid, 9-octadecenoic acid, 9,12-octadecadienoic acid, canophyllal, canophyllol, friedelin, antidesmanol, carpusin, and lupeolactone—and these compounds were also subjected to in silico molecular-docking experiments [19,24,25].

Few scientific reports on the biologic activity of *A. montanum* and no such pharmacological activities related to pain, inflammation, and pyrexia have yet been testified. Hence, the goal of this research was to distinguish the pharmacological approach of *A. montanum* methanol extract by exploring its potential antioxidant, thrombolytic, anti-inflammatory, analgesic, and antipyretic activities in in vivo and in vitro models as well as in silico molecular-docking analysis to gain better insight into the molecular mechanism at play. Subsequently, further findings are needed to develop new molecules with enhanced therapeutic intensity and without any adverse effects that can act as potential novel antagonists.

## 2. Results

### 2.1. Effect of MEAM on Qualitative Phytochemical Screening

The qualitative phytochemical screening of methanol extract of *A. montanum* (MEAM) leaves revealed the presence of secondary metabolites—notably, alkaloids, flavonoids and flavanols, phytosterols, cholesterols, phenols and polyphenols, terpenoids, fatty acids, gums and mucilages, glycosides, fixed oils, emodines, coumarins, resins, and tannins (Table 1).

### 2.2. Effect of MEAM on Total Phenol and Flavonoid Contents

The total phenol and flavonoid contents of crude extract MEAM leaves (500 μg/mL) were explored and the findings of a quantitative analysis of relevant phytochemicals of antioxidants are depicted in Table 2 together with their regression equations. Phenols are a common molecule of bioactive secondary metabolites and presented a total value of 358.31 ± 2.35 mg GAE/g MEAM, whereas the total flavonoid content of the same extract was 23.76 ± 1.45 mg QE/g MEAM.

### 2.3. Antioxidant Activity

#### 2.3.1. Effect of MEAM on 2,2-diphenyl-1-picrylhydrazyl (DPPH) Activity

**Figure 1:**
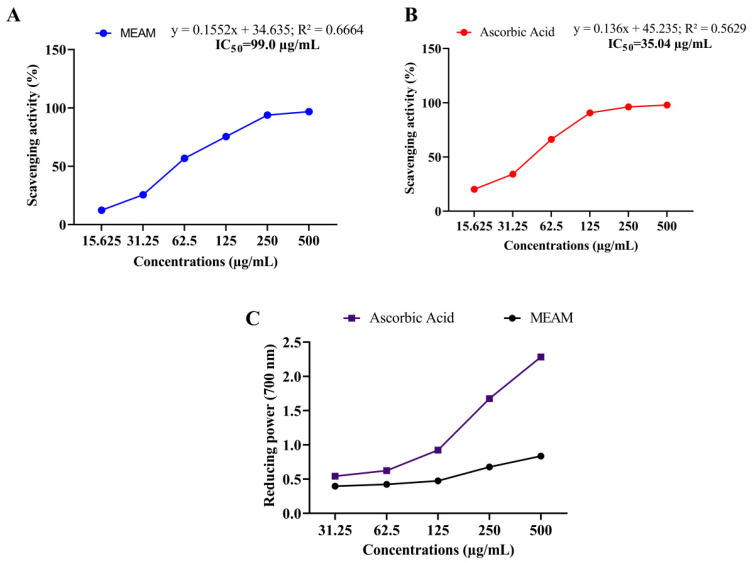
Antioxidant activity of methanol extract of *A. montanum* (MEAM): (**A**) DPPH free radical scavenging activity of MEAM; (**B**) DPPH free radical scavenging activity of ascorbic acid; and (**C**) reducing power capacity of MEAM and ascorbic acid. Values are presented as mean ± S.E.M. (n = 3).

The free radical scavenging activity of MEAM was calculated using the DPPH method given in Figure 1A,B. MEAM’s antioxidant DPPH scavenging activity exhibited significant inhibition (*p* < 0.001) as compared with the standard (ascorbic acid). Meanwhile, the 50% inhibitory concentration (IC_50_) values of the crude extract and ascorbic acid were 35.04 and 99.00 µg/mL.

#### 2.3.2. Effect of MEAM on Ferric-Reducing Power Capacity (FRPC)

Any potential antioxidants are related to electron-transfer activity, which serves as an indicator of a reduction in the power of a compound. A dose–response curve for reducing the MEAM power activity is presented (31.25−500 μg/mL) in Figure 1C, and the maximum absorbance (0.837) was observed at a 500 µg/mL concentration by the spectrophotometer of this assay.

### 2.4. Effect of MEAM on Thrombolytic Activity

**Figure 2:**
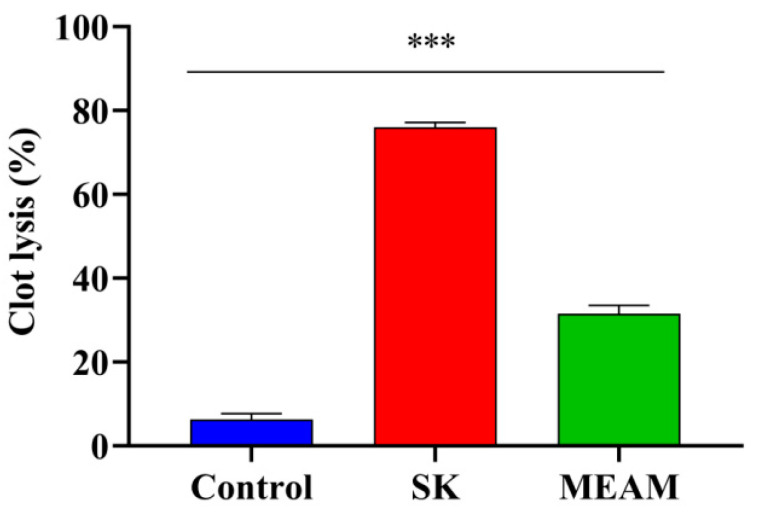
Percentages of clot lysis of in vitro thrombolytic activity of methanol extract of *A. montanum* (MEAM) and standard drug streptokinase (SK). Values are presented as mean ± S.E.M. (n = 5); *** *p* < 0.001 were statistically significant in comparison to control (Dunnett’s test).

The MEAM showed a result of 31.57% (*p* < 0.01) for clot lysis in comparison with that of 6.34% for saline water and 76.00% for streptokinase (Figure 2).

### 2.5. Anti-Inflammatory Activity

#### 2.5.1. Effect of MEAM on Membrane-Stabilization Activity by Human Red Blood Cells (RBCs)

**Figure 3:**
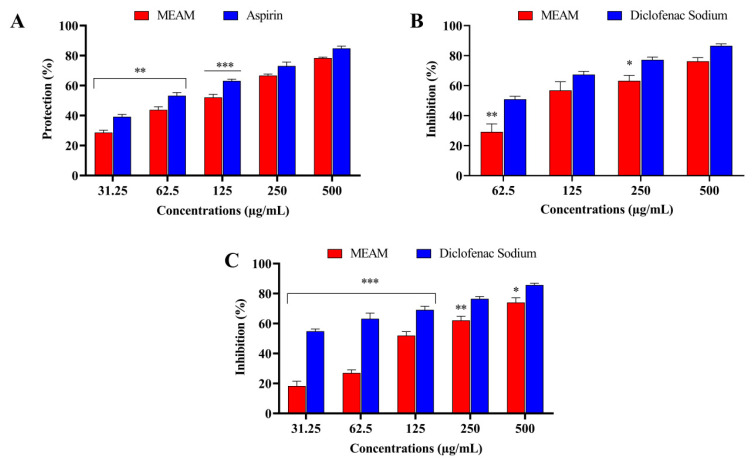
Anti-inflammatory activity of methanol extract of *A. montanum* (MEAM): (**A**) human red blood cell (HRBC) membrane stabilization; (**B**) protein denaturation by using egg albumin; and (**C**) protein denaturation by using BSA. Values are presented as mean ± S.E.M. (n = 5); * *p* < 0.05, ** *p* < 0.01, *** *p* < 0.001, statistically significant in comparison to control (Dunnett’s test).

In the test, the dose-dependent increase in the percentage of inhibition of leaf extract hemolysis was slightly lower than that of the standard (aspirin). The protective result detected with crude extract was 78.36%; moreover, a maximum stabilization value of 84.80% at the highest concentration at 500 μg/mL was attained in aspirin (Figure 3A).

#### 2.5.2. Effect of MEAM on Protein Denaturation Using Egg Albumin

MEAM has shown significant protective activity against the denaturation of egg albumin at several doses (62.5, 125, 250, and 500 μg/mL). The present analysis indicated a significant effect of the crude extract and diclofenac sodium (76.23% and 86.52% of protein-denaturation inhibition), which is displayed in Figure 3B.

#### 2.5.3. Effect of MEAM on the Inhibition of Protein Denaturation Using Bovine Serum Albumin (BSA)

The anti-inflammatory activity triggered by BSA denaturation at different concentrations achieved notable (*p* < 0.001) degrees of inhibition of protein denaturation. In particular, the crude extract produced a 74.05% rate of inhibition at the highest concentration when compared with the standard drug diclofenac sodium (85.75%), as shown in Figure 3C.

### 2.6. Analgesic Activity

#### 2.6.1. Effect of MEAM by Acetic Acid-Induced Writhing Test

**Figure 4:**
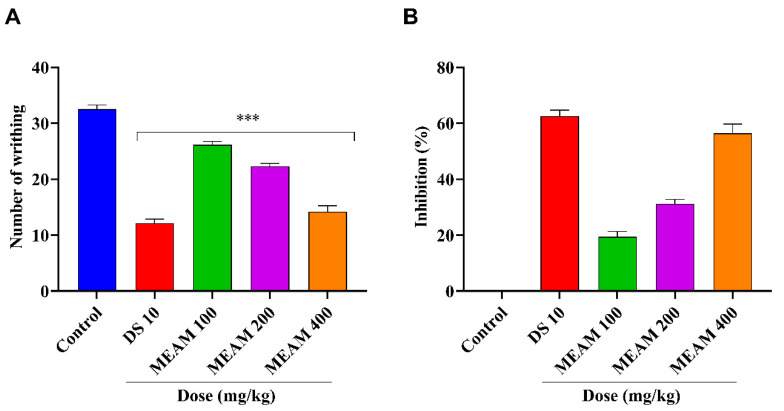
Analgesic effect of methanol extract of *A. montanum* (MEAM) and diclofenac sodium (DS): (**A**) Acetic acid-induced abdominal writhing; and (**B**) percent inhibition. Values are presented as mean ± S.E.M. (n = 6); *** *p* < 0.001, compared with vehicle control (Dunnett’s test).

In this experiment, the MEAM demonstrated a noteworthy (*p* < 0.001) and dose-dependent analgesic response, suppressing the frequency of writhing induced by acetic acid in mice after oral administration. MEAM achieved writhing-inhibition rates of 19.49%, 31.28%, and 56.41% at the mentioned doses, while the standard drug (diclofenac sodium) achieved a 62.56% reduction in pain. These findings are presented in Figure 4.

#### 2.6.2. Effect of MEAM by Formalin-Induced Licking Test

MEAM significantly and dose-dependently triggered a noticeable pain reduction in the biphasic model responses in this study—more specifically, the MEAM extract ensured a remarkable (*p* < 0.001) reduction in pain in both phases at all experimental doses. Thus, the analgesic effects of the MEAM response were correlated with the standard drug (diclofenac sodium), as presented in Table 3.

#### 2.6.3. Effect of MEAM by Tail Immersion Test (TIT)

The response of MEAM administration to this test is summarized in Table 4. During the TIT, the MEAM leaves produced dose-dependence and significantly (*p* < 0.001) distressed the pain-perception response against thermal insult, suppressing the frequency of pain in mice after oral administration. The percentages of the maximal possible effect of both MEAM and diclofenac sodium were significant (*p* < 0.001) at all experimental doses, but the response values of diclofenac sodium varied between the observation periods.

### 2.7. Effect of MEAM by Brewer’s Yeast-Induced Pyrexia

The present research was conceived to test the antipyretic activities of MEAM leaves in vivo. This goal was pursued by subcutaneously injecting brewer’s yeast in each animal with a dose of 10 mL/kg. Brewer’s yeast produces an exogenous pyrogen that causes a pathogenic fever which, during the 4-h study period, remained elevated. After the second hour of therapy, the crude extract achieved a substantial reduction (*p* < 0.001) when using MEAM doses of 200 and 400 mg/kg. A similar protective effect was observed at 3 h of treatment with MEAM treatment at doses of 200 and 400 mg/kg. After 2–4 h, a less-significant antipyretic effect was observed with a dose of 100 mg/kg. Overall, the treated (200 and 400 mg/kg) doses of MEAM comparable to paracetamol (acetaminophen) produced a significant response (*p* < 0.001) after the first hour of treatment and a favorable antipyretic effect throughout the 4-h study period, as reported in Table 5.

### 2.8. In Silico Study

#### 2.8.1. Molecular-Docking Study for Antioxidant Activity

**Figure 5:**
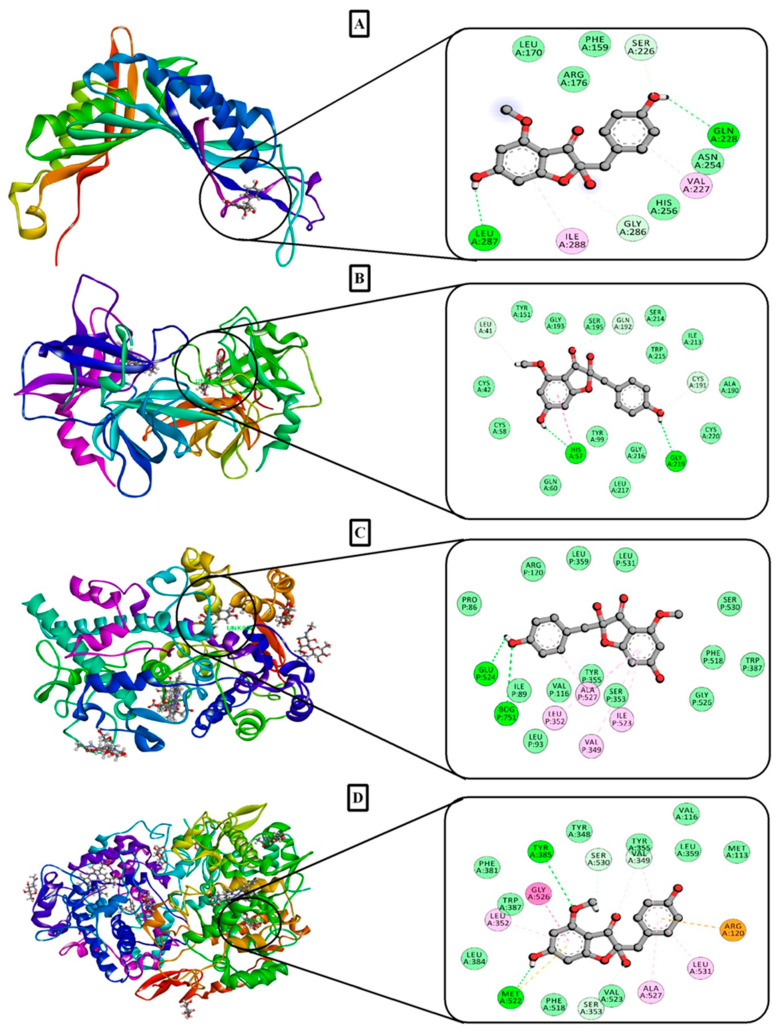
3D and 2D best docking interactions of carpusin with: (**A**) 1R4U; (**B**) 1A5H; (**C**) 2OYE; and (**D**) 6COX.

The molecular-docking simulation results of antioxidant potential are demonstrated in Table 6 and the interactions of the best-docked compound are exhibited in Figure 5A. In this case, the compounds were docked against urate oxidase receptor (PDB ID: 1R4U), where carpusin and 9-ecosyne achieved the maximum and minimum interaction scores of −5.113 and 2.159 kcal/mol, respectively. The order of rank for binding scores is as follows: carpusin (−5.113) > gamma sitosterol (−3.305) > antidesmasol (−2.923) > canophyllol (−2.573) > lupeolactone (−2.506) > canophyllal (−2.298) > friedelin (−2.251) > 3,7,11,15-tetramethyl-2-hexadecen-1-ol (0.07) > tridecanoic acid (1.374) > hexadecanoic acid (1.829) > 9-ecosyne (2.159). Conversely, 9-octadecenoic acid and 9,12-octadecadienoic acid did not exhibit any level of interaction against 1R4U.

#### 2.8.2. Molecular Docking Study for Thrombolytic Activity

In this docking study, carpusin exhibited the highest interaction score (−6.23 kcal/mol) against tissue plasminogen receptor (PDB ID: 1A5H). Carpusin also revealed a stronger binding affinity when compared with the streptokinase (−6.173 kcal/mol). More broadly, the various compounds maintained docking scores in the following ranking order: carpusin (−6.23) > friedelin (−4.241) > canophyllal (−4.228) > antidesmanol (−4.181) > canophyllol (−3.973) > lupeolactone (−3.802) > gamma sitosterol (−3.379) > 9-octadecenoic acid (−2.342) > 3,7,11,15-tetramethyl-2-hexadecen-1-ol (−1.969) > hexadecanoic acid (−1.248) > 9,12-octadecadienoic acid (−0.303) > 9-ecosyne (−0.3) > tridecanoic acid (0.271). These scores are depicted in Table 6 and the candidate with highest binding affinity is shown in Figure 5B.

#### 2.8.3. Molecular Docking Study for Anti-Inflammatory, Analgesic, and Antipyretic Activity

We next sought to evaluate the analgesic, anti-inflammatory, and antipyretic effects, suggesting the COX-1 (PDB ID: 2OYE) and COX-2 (PDB ID: 6COX) abilities. For the COX-1 receptor, the strongest binding affinity was also exhibited by carpusin (−7.613 kcal/mol) which is better than the docking score of the diclofenac sodium (−7.545 kcal/mol). The other candidates with docking interactions with the COX-1 receptor achieved the following ranking order: 3,7,11,15-tetramethyl-2-hexadecen-1-ol (−2.941) > 9-ecosyne (−2.371) > 9,12-octadecadienoic acid (−2.229) > 9-octadecenoic acid (−2.107) > hexadecanoic acid (−1.663) > tridecanoic acid (−0.822). In this experiment, gamma sitosterol, friedelin, canophyllol, canophyllal, antidesmasol, and lupeolactone did not interact with the COX-1 receptor at all. For the COX-2 receptor, carpusin exhibited the best binding interaction of −8.678 kcal/mol, which was higher than of the standard, diclofenac sodium (−6.917 kcal/mol), while the other compounds maintained the following order regarding binding interactions with COX-2: antidesmasol (−4.758) > canophyllol (−3.763) > 3,7,11,15-tetramethyl-2-hexadecen-1-ol (−3.761) > 9,12-octadecadienoic acid (−3.127) > canophyllal (−2.099) > 9-octadecenoic acid (−0.768) > tridecanoic acid (−0.768) > hexadecanoic acid (0.182) > 9-ecosyne (0.318). Gamma sitosterol, friedelin, and lupeolactone did not show any type of binding affinity with the COX-2 receptor. These scores are displayed in Table 6 and the binding interactions of the best-docked compounds are displayed in Figure 5C,D.

#### 2.8.4. Absorption, Distribution, Metabolism, Excretion, and Toxicity Analysis (ADME/T) and Toxicology Properties Prediction

The ADME/T properties of the isolated compounds of MEAM are exhibited in Table 7. As per Lipinski’s rule of five, tridecanoic acid and carpusin met all of the conditions considered to suggest the existence of good oral bioavailability and drug-like characteristics. Meanwhile, the rest of the compounds did not meet more than two conditions, so they can be considered as safe. In addition, a toxicological properties prediction performed using the admetSAR online server exposed the fact that none of the constituents possess Ames toxicity or carcinogenicity (except 9-ecosyne) and appeared to have only weak rat acute toxicity characteristics, as shown in Table 8.

## 3. Discussion

In recent times, plant-derived substances have garnered significant interest because of their remarkable applications [26]. Medicinal plants are the richest bioresources of conventional drugs, modern medicines, nutraceuticals, curative intermediates, and artificial drug chemicals. Phytochemical screening has revealed the existence of both physiological and therapeutic activities [27]. In our recent research, *A. montanum* (Pani heloch) leaves exhibited the presence of secondary metabolites—notably, flavanols, flavonoids, phenols, alkaloids, polyphenols, terpenoids, emodines, glycosides, coumarins, cholesterols, resins, tannins, gums and mucilages, phytosterols, fatty acids, and fixed oils [28].

Plants are a magnificent source of natural antioxidants to ensure the safety of free radicals against oxidative stress [29]. MEAM leaves were examined for antioxidant activity using two usually acknowledged techniques: DPPH and ferric-reducing power analysis. Of note, it was determined that plant extractives reduced the stable DPPH radical content and minimized color changes due to the presence of antioxidant properties in samples [30]. The extract showed notable (*p* < 0.001) scavenging activity at the highest concentration (500 µg/mL) as compared with ascorbic acid, whereas the IC_50_ value of the standard and crude extracts were 35.04 and 99.00 µg/mL, respectively. Therefore, flavonoids, phenols, and tannins can be immensely important compounds for the free radical scavenging mechanism of extractives [31,32].

Results of the ferric reduction assay of MEAM leaves were calculated using the method of the reduction of potassium ferric cyanide. This approach relies on the reduction of Fe^3+^ to Fe^2+^ by changing the color of the test solution using antioxidant compounds. Notably, the crude methanol extract showed the highest ferric reduction potential among the leaf extracts tested in this study. Phenol-presenting compounds may play an important role in the reduction capacity of the extracts; the present quantitative investigation found the highest amount of phenol compounds, which may be one of the causes and a significant indicator of potential antioxidant activity [33].

Various researchers have attempted to confirm antithrombotic effects in plant sources and their supplements to prevent coronary disease [26,34]. Tissue plasminogen activator, urokinase, and streptokinase are commonly used in the treatment of thrombotic disorders. Drugs are available on the market that convert plasminogen to plasmin and remove clot lysis [35,36]. Clot lysis is also useful for treating clot disorders, including myocardial infarction, thromboembolic stroke, deep vein thrombosis, and pulmonary embolism [37]. Both MEAM and streptokinase exhibited a moderate degree of clot lysis in comparison with in the control group as depicted by our recent findings. The leaves of MEAM were previously reported [24] to contain some terpenoids as phyto-constituents, which might be the reason for this response.

Inflammation is a response to tissue injury and a complex process of enzymatic reaction [38]. However, the analysis of erythrocyte (RBC membrane) membrane stabilization is a standard method for screening for anti-inflammatory properties [39,40]. The lysosomal membrane is comparable to the erythrocyte membrane and its stabilization also serves as a parameter suggesting an extract’s ability to stabilize the membrane. In addition, the strength of erythrocytes (RBCs) is dependent upon the integrity of their membranes and the exposure of erythrocytes to the hypotonic medium leads to lysis of said membranes [41]. The membrane-stabilizing impute of *A. montanum* could be responsible for its restraining and protective action on the release of neutrophils’ lysosome content as an explicit indicator of anti-inflammatory activity [42]. In this assessment, MEAM appeared to show the maximum (*p* < 0.001) membrane stabilization capacity at the highest concentration as compared with the standard (aspirin). The secondary metabolites containing phenols and tannins might be responsible for this activity.

Meanwhile, protein denaturation is caused by an inflammatory process, often arthritis [43]. It can be protected against by monitoring their inhibitory response on COX, which assumes a significant role in the management of inflammation. To assess the anti-inflammatory activity, one should select the convenient method of anti-denaturation assay, which is carried out when the secondary and tertiary protein structures are lost by heat, organic solvent, acid, or base and some are thereafter revealed as extrinsic factors [44]. In the production of autoantigens in certain rheumatic ailments, protein denaturation might be expected. MEAM leaves can be applied by inducing heat treatment; in this study, the anti-denaturation approach incorporating egg albumin was chosen. The denatured protein exhibits the antigen associations of a Type III hypersensitive response, while denatured proteins induced by heat are more effective than native proteins in fostering delayed hypersensitivity [39]. Moreover, traditional NSAIDs such as phenylbutazone and indomethacin have already been shown to function not only by inhibiting the development of endogenous prostaglandins by blocking the COX enzyme but also by inhibiting protein denaturation [8]. However, alterations of electrostatic, hydrogen, hydrophobic, and disulfide bonding are involved, probably due to the mechanism of denaturation [41]. In our recent assessment, MEAM inhibited heat-induced protein denaturation as measured with the standard, which may be the possible reason for the observed anti-inflammatory and anti-arthritic activities realized by controlling the autoantigen production. A preliminary phyto-constituent study of MEAM indicated flavonoids, terpenoids, tannins, and phenols as commonly responsible for these pharmacological responses [45].

The assessment of MEAM’s in vivo analgesic effect was performed at three separate doses (100, 200, and 400 mg/kg) using chemical (acetic acid and formalin test) and thermal (TIT) models of pain in mice. An antinociceptive or painkiller drug was used to reduce pain [46]. The active component of the plant showed some potential activities similar in scope to antioxidant properties, which may suggest the antinociceptive effect of the extract. Meanwhile, the writhing reaction caused by acetic acid is a responsive mechanism for evaluating peripherally-acting analgesics and reflects pain by inducing a localized inflammatory response due to the release of free arachidonic acid from tissue phospholipid [47,48]. This reaction is thought to be mediated by the acid-sensing ion channels of peritoneal mast cells and the prostaglandin pathways. Besides, organic acid is also thought to function indirectly to trigger the release of endogenous mediators that activate nociception [49,50]. It is notable that NSAIDs and pain-relieving drugs act by preventing moderate degrees of pain with the formation of antinociceptive signals that block the synthesis of prostaglandins, tumor necrosis factor-α, and bradykinin [51]. During the writhing test, MEAM and the standard drug diclofenac sodium showed a significant (*p* < 0.001) and dose-dependent number of writhing events as compared with in the control group. Moreover, the extract’s MEAM suppression of neurogenic and inflammatory pain during the formalin test indicated that it may contain active principle metabolites that can function both centrally and peripherally. Moreover, our results support that the mechanism by which formalin triggers C-fiber activation has remained unknown for a comparatively long time [52]. Besides, the formalin-induced assessment in a mouse model of methanol extract can reduce the pain in both the early and late phases as compared with the standard drug. Tail-immersion testing (TIT) against a thermal threshold in mice exposed an increased latency time; this result was observed in the case of both extracts and the standard drug. The present study further suggests that MEAM possesses a significant (*p* < 0.001) analgesic effect linked to both central and peripheral mechanisms and provides a rationale for its extensive use at various painful conditions. Consequently, the finding of MEAM exhibiting a significant analgesic effect on mice may be the result of the presence of alkaloids and flavonoids. 

Pyrexia is a secondary effect of tissue damage and may accompany pain, discomfort, irritation, malaria, malignancy, or other diseases [53]. Brewer’s yeast and D-amphetamine are widely used in mice for the activation of pyrexia [54] due to the presence of proteins containing yeast [53,55]. Among other mediators, prostaglandin-synthesis inhibition may be observed as a potential antipyretic mechanism that blocks the activity of the COX enzyme, enhances the antipyretic message in the brain, and activates anti-inflammatory signals at the injury site [56]. Besides, the methanol extract significantly lowered the temperature in yeast-induced pyrexia to correlate with the reference drug, paracetamol. Therefore, the plant has antipyretic effects that are mediated by prostaglandin-synthesis interference and cytokinase-release inhibition due to the existence of steroids, alkaloids, and flavonoids [53].

A computational molecular-docking study is an essential method for the prediction of the binding capacity of active biological constituents against selected proteins [57,58]. A molecular-docking study was conducted to support the results of this study, where carpusin (−6.23 kcal/mol) showed excellent and friedelin (−4.241 kcal/mol), canophyllal (−4.228 kcal/mol), antidesmanol (−4.181 kcal/mol), canophyllol (−3.973 kcal/mol), and lupeolactone (−3.802 kcal/mol) showed moderate docking scores, respectively. Additionally, 13 compounds were docked against four target receptors and, among all the examined compounds, carpusin showed a better binding with each receptor than the reference drug. Moreover, ADME/T analysis of carpusin demonstrated that it adheres to the Lipinski’s rule of five and does not possess any toxicity, including Ames toxicity, carcinogenicity, acute oral toxicity, and rat acute toxicity. Accordingly, carpusin can be considered as a promising drug candidate due to its good oral bioavailability and multiple pharmacological actions. However, subsequent investigation is needed to isolate the compounds as pure forms and to understand the molecular mechanism to ensure long-term safety. Besides, the significant results of anti-inflammatory, analgesic, and antipyretic potentials may conceivably be because of the presence of carpusin as a bioactive compound in MEAM. Carpusin appeared to have the strongest docking interaction against COX-1 (−7.613 kcal/mol) and COX-2 (−8.678 kcal/mol) receptors, with higher scores than those attained by the diclofenac sodium (COX-1: −7.545 kcal/mol; and COX-2: −6.917 kcal/mol).

## 4. Materials and Methods

### 4.1. Plant Collection and Identification

Fresh leaves of Pani heloch (*Antidisma montanum* Blume) were collected in September 2019 at the Botanical Garden and Eco Park (22°36′00″ N, 91°40′14.13″ E) in Sitakunda, Chittagong, Bangladesh, then identified and authenticated by Dr. Shaikh Bokhtear Uddin, Professor and Taxonomist in the Department of Botany, University of Chittagong in Chittagong, Bangladesh. The voucher was kept at the University Herbarium with plant samples (access number: SBU.29153).

### 4.2. Extraction Process

The collected leaves were thoroughly washed and shade-dried for one week with a suitable temperature (60 °C) maintained, then crushed into a coarse powder using an automatic NOWAKE grinder (NOWAKE, Hokuto, Japan). Thereafter, the coarse leaf powder (800 g) was collected in a very clean flask and immersed in 3 L of methanol for 12 days, with shaking and stirring periodically, at room temperature. In addition, the mixture was initially filtered by Whatman No. #1 filter paper application with a cotton plug. Then the filtrates were evaporated by rotary evaporator at 45 °C and dried to obtain a semisolid extract (30 g, 3.75% *w*/*w*). The crude extract was refrigerated until further analysis at 4 °C.

### 4.3. Drugs and Chemicals

The following analytical-grade reagent chemicals were used in all experiments: quercetin, DPPH, diclofenac sodium, gallic acid, and aspirin (Sigma-Aldrich, Chemical Co., St. Louis, MO, USA). Folin–Ciocalteu reagent, sodium carbonate, and methanol were purchased from Merck (Darmstadt, Germany). BSA was obtained from SD Fine Chem. Ltd. (Mumbai, India). Acetic acid and formalin were attained from Merck, whereas streptokinase (1,500,000 IU) was acquired from Beacon Pharmaceuticals Ltd. (Bangladesh, India).

### 4.4. In Vitro Pharmacological Study

#### 4.4.1. Qualitative Phytochemical Screening

The qualitative phytochemical screening of MEAM leaves to evaluate the alkaloids, flavonoids, flavanols, phytosterols, cholesterols, phenols, polyphenols, terpenoids, saponins, amino acids, fatty acids, starches, gums and mucilages, carboxylic acids, glycosides, fixed oils, emodine, carbohydrates, proteins, steroids, anthocyanins, coumarins, resins, and tannins was conducted by using standard phytochemical procedures [8,59], which are briefly presented in the Supplementary Materials.

#### 4.4.2. Quantitative Phytochemical Analysis

##### Determination of the Total Phenol Content (TPC)

The TPC of the extract was depicted by using Folin–Ciocalteu reagent, adapted from a published method with some amount of modification [60]. Gallic acid was used as a standard drug in this step. Briefly, 2.5 mL of Folin–Ciocalteu reagent (10 times diluted) and 2.5 mL of 20% Na_2_CO_3_ were combined with crude extract (500 μg/mL). The mixture was then brought up to 10 mL by adding distilled water, then incubated to complete the reaction for 20 min at 25 °C; subsequently, the absorbance was measured at 765 nm. Thereafter, the TPC concentration of the extract was calculated by the equation acquired from the standard gallic acid curve (mg GAE/g extract) as an equivalent of gallic acid.

##### Determination of Total Flavonoid Content (TFC)

The standard colorimetric method was used to identify the TFC of the extract, while quercetin was used as a standard [61,62]. In this, 1.5 mL of methanol and 100 μL of AlCl_3_ (10%) were mixed with the extract (500 μg/mL). Then, 100 μL of potassium acetate (1 M) and 2.8 mL of distilled water were added to the mixture, which was placed into the incubator to complete the reaction for 30 min at room temperature. Besides, the absorbance was measured at 415 nm. Thus, the total flavonoid content was determined, which correlated with the quercetin-equivalent concentration (mg QE/g MEAM).

#### 4.4.3. Antioxidant Activity

##### DPPH Antioxidant Scavenging Assay

MEAM’s free-radical scavenging activity was determined by using the DPPH method for discerning the radical scavenging capacity [63]. Briefly, 3 mL of 0.004% DPPH solution (4 mg of DPPH in 100 mL of 95% methanol) was applied in conjunction with various raw extract and standard drug concentrations (15.625, 31.25, 62.5, 125, 250, and 500 µg/mL). The concentrate was homogenized and incubated for 30 min at room temperature in the dark and a spectrophotometer was used to measure the absorbance at 517 nm [64]. The following equation was used to calculate the percentage radical scavenging activity:

$$\mathrm{Radical}\;\mathrm{scavenging}\;\left(\%\right)\;=\;\frac{\left(\mathrm{Ac}\;–\;\mathrm{As}\right)}{\mathrm{Ac}}\times 100$$

where Ac is the absorbance of the control and As is the absorbance of the standard or extract. The IC_50_ was also calculated as it indicated the effective concentration of the extract needed to scavenge 50% of the free radicals of DPPH.

##### FRPC Test

The FRPC test outcome of MEAM was assessed by the described method with some modifications [65]. Briefly, 1 mL of test solution (extract) and ascorbic acid were added in different concentrations (31.25, 62.5, 125, 250, and 500 µg/mL) with 2.5 mL of phosphate buffer and 2.5 mL of 1% *w/v* potassium ferricyanide, respectively. The mixture was implanted for 20 min at 50 °C to complete the reaction, and then trichloroacetic acid (2.5 mL) solution was added, followed by centrifugation (3000 rpm) for 10 min. Next, the supernatant solution (2.5 mL) was withdrawn from this mixture and 2.5 mL of distilled water and FeCl_3_ were added, respectively. Finally, absorbance was taken at 700 nm, whereas ascorbic acid was treated as the standard.

### 4.5. Thrombolytic Assay

The clot lysis study was performed with a certain level of modification according to the described method [66,67]. As a stock solution, one vial of lyophilized streptokinase (1,500,000 IU) was adequately mixed with 5 mL of sterilized distilled water, resulting in optimal dilution. Subsequently, 5 mL of venous blood were collected from healthy volunteers (n = 5), who are normal BMI with the age of 22–25 years, non-smokers, and did not take any oral anticoagulant or contraceptive for about 10 days, then delivered in sterile preweighted Eppendorf tubes (0.5 mL/tube). The freshly collected blood was labeled correctly and incubated at 37 °C for 45 min, which allowed for the formation of clots. After clot formation, the serum was completely withdrawn without damaging the clots and each tube was weighed again. Next, 100 µL of extract (100 mg/10 mL) was added to each Eppendorf tube containing preweighed clots. Streptokinase (positive control) and distilled water (negative control) were also added separately to the preweighted Eppendorf tubes with clots and incubated for 90 min at 37 °C, then removed, and the tube was reweighed to obtain the primary and final weights required to confirm clot lysis (thrombolysis) had been achieved. This experiment was performed three times with the same blood samples of five volunteers and the results were determined using the following equation:

 Clot lysis (%)= (wt. of released clot/clot wt.)×100

### 4.6. Anti-Inflammatory Assay

#### 4.6.1. Membrane Stabilization by Human RBCs

The present study protocol was developed with certain modifications of the underlying method [68], which was used to depict in vitro determination of the membrane-stabilizing activity. In short, 5 mL of blood were taken from healthy human volunteers for the preparation of erythrocyte suspension, which was combined with the equivalent sterile volume. MEAM extract was added with Alsever’s solution and centrifuged (3000 rpm) for 15 min. The filled cells were then washed three times with isosaline and a suspension was prepared (10% *v*/*v*), which was used for this analysis [69]. The test solution was composed of 1 mL of phosphate buffer, 2 mL of hypotonic saline, and 0.5 mL of MEAM prepared at various concentrations (31.25, 62.5, 125, 250, and 500 μg/mL), whereas the control solution included 1 mL of phosphate buffer, 2 mL of distilled water, and 0.5 mL of human RBC in isotonic saline, while aspirin was adopted as a standard. The stirred mixture was applied for 30 min at 37 °C and centrifuged (3000 rpm) for 15 min. Additionally, the supernatant of the mixtures was decanted and a spectrophotometer was used to calculate the hemoglobin content at 560 nm. The test was performed in triplicate and the hemolysis percentage was estimated using the following formula:

$$\;\mathrm{Haemolysis}\;\left(\%\right)\;=\;\frac{\mathrm{OD}\;\mathrm{of}\;\mathrm{test}}{\mathrm{OD}\;\mathrm{of}\;\mathrm{control}}\;\times 100$$

Then, the percentage of human RBC membrane stabilization or protection was calculated by using the following formula:

Protection (%)= 100−(OD of test)/(OD of control) ×100

where OD is the optical density.

#### 4.6.2. Anti-Inflammatory Test with the Inhibition of Protein Denaturation by Egg Albumin

The anti-inflammatory effect of MEAM was tested using albumin-denaturation inhibition techniques according to an earlier approach with contemptible modifications [8]. The reaction mixture (5 mL) was composed of 0.2 mL of fresh hen’s egg albumin, 2.8 mL of phosphate-buffered saline, and 2 mL of various extracts to reach final concentrations of 62.5, 125, 250, and 500 μg/mL, respectively. At that point, mixtures were hatched at 37 ± 2 °C in a biological oxygen demand (BOD) incubator for 15 min and then heated at 70 °C for 5 min. After that, the absorbance values of the prepared sample, vehicle (water), and reference drug (diclofenac sodium) were determined at 660 nm (UV 1800; Shimadzu Corporation, Kyoto, Japan). The test was performed in triplicate and the percentage of inhibition of protein denaturation was determined using the following equation:

$$\mathrm{Inhibition}\;\left(\%\right)=\frac{\mathrm{Abs}\;\mathrm{control}\;-\;\mathrm{Abs}\;\mathrm{test}}{\mathrm{Abs}\;\mathrm{control}}\times 100$$

#### 4.6.3. Anti-Inflammatory Test with the Inhibition of Protein Denaturation by BSA 

The anti-inflammatory effect of MEAM was investigated using a protein-denaturation inhibition technique based on a previous approach with minor modifications [70]. The reaction mixture (0.5 mL) consisted of BSA (0.45 mL), MEAM (0.05 mL), and standard solution at different concentrations (31.25, 62.5, 125, 250, and 500 µg/mL), while diclofenac sodium was used as a standard. The sample was hatched for 20 min at 37 °C and again retained for 30 min at 57 °C. In addition, 2.5 mL of phosphate buffer were applied to the previous solutions and spectrophotometer absorption was estimated at 660 nm, with distilled water used as a test control. The test was conducted in triplicate and the percentage of protein-denaturation inhibition was calculated as follows:

$$\;\mathrm{Inhibition}\;\left(\%\right)\;=\frac{\mathrm{Abs}\;\mathrm{control}\;-\;\mathrm{Abs}\;\mathrm{test}}{\mathrm{Abs}\;\mathrm{control}}\times 100$$

### 4.7. In Vivo Pharmacological Study of MEAM Leaves

#### 4.7.1. Experimental Animals and Ethical Statements

From the International Diarrheal Disease Research Center in Dhaka, Bangladesh, we procured adult Swiss albino mice with weights ranging from about 20–25 g. The experimental animals were delivered with standard laboratory food and portable water and were exposed to adequate ventilation in the experiment room during the natural daylight period. All experiments were performed in an isolated and silent state according to the eighth edition of the United States Laboratory Animal Care and Use guidelines [71]. The protocol of the study was approved by the Institutional Animal Ethical Committee, the Research and Development Committee (IIUC/PHARM-AEC-147/13-2019); and the Department of Pharmacy of the International Islamic University Chittagong, Bangladesh. The animals were adjusted for 10 days to the conditions of the laboratory before experimentation.

#### 4.7.2. Acute Oral Toxicity Test

The acute oral toxicity test was performed by following a standard method [72]. Twenty-one Swiss albino male mice (25–30 g) were used for the acute toxicity test and divided into five groups, where each group contained six mice and different doses of the MEAM leaves (50, 100, 200, 400, or 2000 mg/kg body weight) were administered by gavage for observation. Mice were kept fasting overnight before administering the extract and food was also delayed for 3–4 h. All research animals were tracked separately for the next 72 h, focusing on their behavioral changes, allergic syndromes (itching, flatulence, skin rash), and mortality, with precise monitoring of potential irregular reactions.

### 4.8. Experimental Design

The analgesic activity was assessed through paw licking, writhing, and TIT experiments following the treatment of mice, which were divided into five groups of six mice each. The mice were Swiss albino (25–30 g) and all mice were left unfed for two hours prior to the start of the test. Administration of the vehicle occurred in Group I (1% Tween-80 solution) mice; Group II mice were administered the standard drug, diclofenac sodium (10 mg/kg) solution; and MEAM extract (100, 200, and 400 mg/kg) was administered to Groups III, IV, and V mice orally. Additionally, the antipyretic activity subgroup of Group I received Tween-80 solution as a negative control; that of Group II received paracetamol 100 mg/kg (standard); and those of Groups III, IV, and V were subjected to MEAM at the doses of 100, 200, and 400 mg/kg, respectively.

#### 4.8.1. Analgesic Activity of MEAM leaves

##### Acetic Acid–Induced Writhing Test

The experiment followed a previous method described, with some level of modification [73]. After 30 min of the administration of the selected dose to each group of mice, the mice were given an intraperitoneal injection (0.7% acetic acid solution *v*/*v*) to trigger writhing. After treatment, the mice were put separately in transparent cages, allowed to escape for five minutes, and then acid-induced writhes were counted for 20 min. The analgesic effect was demonstrated as a percentage of the inhibition of pain and calculated using the following equation:

$$\;\mathrm{Pain}\;\mathrm{inhibition}\;\left(\%\right)\;=\frac{\mathrm{Wc}-\mathrm{Wts}}{\mathrm{Wc}}\times 100$$

where Wc is the mean number of writhing events in the control group and Wts is the mean number of writhing events in the test sample.

##### Formalin-Induced Licking Test

The experiment followed a previous method described with some modification [73,74]. The right hind paws of all mice were treated by subcutaneous injection of 20 μL of 2.5% (*v*/*v*) formalin solution prepared in distilled water to induce pains. Then, the duration (in seconds) of reaction time (paw licking or biting) was assessed between the initial phase (0–5 min, neurogenic) and the second phase (15–30 min, inflammatory pain) after formalin insertion. Analgesic activity was expressed and measured using the following formula as the percentage of inhibition of paw-licking time:

$$\mathrm{Inhibition}\;\left(\%\right)=\frac{\mathrm{Lc}-\mathrm{Lts}}{\mathrm{Lc}}\times 100$$

where Lc is the licking time of the control group in seconds and Lts is the licking time of the test sample in seconds.

##### Tail Immersion Test (TIT)

The TIT, which involved inducing pain by thermal means, adhered to a previously established method with a minor level of modification [75]. In short, a 1000-mL beaker full of hot water was maintained at a suitable temperature (55 ± 0.5 °C) for the tail dipping of mice. After respective doses (100, 200, and 400 mg/kg) were administered, 1–2 cm of each mouse’s tail was submerged in hot water and a response to pain was considered to have occurred following the animal’s immediate removal of their tail from hot water. The maximum possible response time effect was recorded as the latent duration of tail immersion and deflection at zero, 30, 60, 90, and 120 min after the administration of all test samples (100, 200, and 400 mg/kg), which correlated with the control group. A cumulative submersion time of 20 s was utilized to avoid tail tissue impairments in mice. The percentage of elongation was determined using the following formula:

$$\mathrm{Elongation}\;\left(\%\right)=\;\frac{\mathrm{Latency}\;\mathrm{of}\;\mathrm{test}\;\mathrm{animal}-\mathrm{Latency}\;\mathrm{of}\;\mathrm{control}\;\mathrm{animal}}{\mathrm{Latency}\;\mathrm{of}\;\mathrm{control}\;\mathrm{animal}}\;\times 100$$

#### 4.8.2. Antipyretic Activity by Brewer’s Yeast-Induced Pyrexia

This experiment followed a previously established method with minor modification [10,76]. The normal rectal temperature of each mouse and that following the subcutaneous injection of 20% of the Brewer’s yeast aqueous suspension in all mice to induce pyrexia (treatment Groups III–V) were recorded using a digital thermometer. After 24 h, the rectal temperature of each mouse was reported and those mice with an increase in the body temperature of at least 0.5–1 °C were selected for the experiment. The rectal temperature was recorded again frequently at 1, 2, 3, and 4 h after all the administration of the test sample (MEAM 100, 200, and 400 mg/kg). To assess the activity of each sample, the treated extract groups were compared with the control groups (Groups I and II).

### 4.9. In Silico Study

#### 4.9.1. Molecular Docking Study

##### Preparation of Ligand

The component structures acquired from MEAM were downloaded from the PubChem compound database. Using Schrödinger Suite-Maestro v 11.1′s LigPrep method, the structures in Epik 2.2 were prepared at pH 7.0 ± 2.0 and minimized by force field OPLS3.

##### Preparation of Protein

3D crystal structures of selected receptor proteins were retrieved in PDB (Protein data bank) format [77], namely urate oxidase receptor (PDB ID: 1R4U) [78], tissue plasminogen activator receptor (PDB ID: 1A5H), the receptor cyclooxygenase-1 (PDB ID: 2OYE) [79], the receptor cyclooxygenase-2 (PDB ID: 3HS5) [80]. Using the Protein Preparation Wizard of the Schrodinger Maestro v11.1, the crystal structures of the proteins were optimized. It assigned bond orders, attached hydrogens to heavy atoms and converted selenomethionines into methionines. Removing the water molecules, minimization of heavy atom molecule was performed at RMSD (0.30 Å) by using force field OPLS3.

##### Glide Standard Precision Docking

Standard precision docking was carried out using Glide of Schrodinger Suite-Maestro v 11.1 [81]. To generate the receptor grid for interaction between protein and ligand all parameters were set in defaults and OPLS3 was used as force field. Covering the active site residues of individual receptor a cubic box of definite dimensions was generated. The best fit score for each interaction was noted as docking score [82].

#### 4.9.2. ADME Analysis

Determination of drug-likeness or pharmacokinetic properties of the constituents from *A. montanum* were determined using SwissADME online tool [83]. Based on Lipinski’s rule of five [84], a compound should not violated more than two of the following parameters: (i) molecular weight (acceptable range: ˂500); (ii) high lipophilicity (expressed as LogP, acceptable range: <5); (iii) hydrogen bond acceptor (acceptable range: ≤10); (iv) hydrogen-bond donors (acceptable range: ≤5); and (v) molar refractivity should be between 40 and 130.

#### 4.9.3. Toxicological Properties Prediction by admetSAR

By using admetSAR online tool [85], the toxicological characteristics of the selected constituents were evaluated since safety of consumption depends on the toxicity level. The investigation was determined the acute oral toxicity, carcinogenicity, Ames toxicity and rat acute toxicity.

### 4.10. Statistical Analysis

Data were calculated by using GraphPad Prism version 8.4., and all the values were stated as mean ± S.E.M. (standard error mean), where the values were evaluated as significantly different at *** p < 0.001, ** p < 0.01, and * *p* < 0.05, with one-way analysis of variance (ANOVA) (Dunnett’s test), whereas two-way ANOVA with repeated measures was used for anti-inflammatory and formalin induced licking test. The in vitro study implemented triplicate measurements, with six mice per group for the in vivo model.

## 5. Conclusions

The interpretation of the present scientific research results reflect that a constituent of plants possesses biologically active potential compounds with antioxidant, thrombolytic, and anti-inflammatory activities due to the presence of secondary metabolites—namely, alkaloids, glycosides, and flavonoids. In addition, the outcome of the present research of MEAM (Pani heloch) showed potential analgesic and antipyretic activities that may suppress inflammatory mediators such as tumor necrosis factor-α, nuclear factor κB, COX-1, and COX-2. Thus, this contemporary research can offer pharmacological evidence for the folkloric use of Pani heloch leaves and reveals strongly that the methanol extract contains some active biomarkers that may be responsible for these activities.

## Figures and Tables

**Table 1:** Qualitative phytochemical analysis of MEAM leaves.

Name of the Phytochemicals	MEAM	Name of the Phytochemicals	MEAM
Alkaloids	++	Carboxylic acids	−
Flavonoids and flavanols	++	Coumarins	++
Proteins	−	Starch	−
Cholesterols	+	Anthocyanins	−
Resins	+	Gums and mucilage’s	+
Phenols and polyphenols	++	Phytosterols	+
Terpenoids	++	Carbohydrates	−
Steroids	−	Amino acids	−
Emodines	++	Fatty acids	+
Tannins	+	Saponins	−
Glycosides	++	Fixed oils	+

++ present in moderately high concentration; + present in low concentration; − not present; MEAM, methanol extract of *Antidesma montanum*.

**Table 2:** Quantitative analysis of antioxidant relevant phytochemicals containing total phenol content and total flavonoid content of MEAM leaves.

Treatment	Total Phenol Content (mg GAE/g MEAM)	Total Flavonoid Content (mg QE/g MEAM)
MEAM	358.31 ± 2.35	23.76 ± 1.45
R. EQ.	y = 0.0187x + 0.062; R^2^ = 0.9979	y = 0.0096x − 0.0234; R^2^ = 0.9602

R. EQ., Regression equation.

**Table 3:** Analgesic effect of methanol extract of *A. montanum* (MEAM) and diclofenac sodium (DS) in formalin-induced paw licking test in mice.

Treatment (mg/kg)	Early Phase	Inhibition (%)	Late Phase	Inhibition (%)
Control	40.56 ± 0.52	-	34.33 ± 0.87	-
DS 10	14.68 ± 0.71 ***	63.81	11.51 ± 0.53 ***	66.47
MEAM 100	34.66 ± 0.94 ***	14.55	29.65 ± 0.79 ***	13.63
MEAM 200	30.63 ± 0.69 ***	24.48	22.89 ± 1.02 ***	33.32
MEAM 400	19.54 ± 0.96 ***	51.82	14.76 ± 0.73 ***	57.0

*** *p* < 0.001, compared with vehicle control (Dunnett’s test).

**Table 4:** Analgesic effect of methanol extract of *A. montanum* (MEAM) and diclofenac sodium (DS) on tail immersion test in mice.

Treatment (mg/kg)	Response Times (s) (% MPE)				
	0 min	30 min	60 min	90 min	120 min
Control	3.13 ± 0.05	4.22 ± 0.15	4.25 ± 0.06	3.97 ± 0.06	3.83 ± 0.06
DS 10	3.34 ± 0.03	6.27 ± 0.16 *** (17.59)	7.49 ± 0.15 *** (24.93)	7.55 ± 0.24 *** (25.27)	6.82 ±0.05 *** (20.876)
MEAM 100	3.16 ± 0.04	3.95 ± 0.05 (4.75)	4.99 ± 0.04 *** (10.90)	5.34 ± 0.16 *** (12.96)	5.08 ± 0.09 *** (11.38)
MEAM 200	3.17 ± 0.09	4.22 ± 0.17 (6.27)	5.68 ± 0.19 *** (14.89)	5.73 ± 0.17 *** (15.23)	5.74 ± 0.32 *** (15.32)
MEAM 400	3.13 ± 0.05	5.35 ± 0.16 *** (13.15)	6.57 ± 0.15 *** (20.41)	7.36 ± 0.10 *** (25.09)	6.93 ± 0.14 *** (22.58)

MPE, maximal possible effect. *** *p* < 0.001, compared with vehicle control (Dunnett’s test).

**Table 5:** Antipyretic effect of methanol extract of *A. montanum* (MEAM) on Brewer’s yeast induced pyrexia in mice.

Treatment (mg/kg)	Basal Temp. (°F)	0 Hour (after 24 Hour)	Rectal Temperature (°F)			
			1st Hour	2nd Hour	3rd Hour	4th Hour
Control	99.20 ± 0.16	101.02 ± 0.15	100.82 ± 0.11	100.61 ± 0.09	100.43 ± 0.11	100.20 ± 0.17
Paracetamol 100	98.82 ± 0.21	100.50 ± 0.16 *	99.98 ± 0.14 ***	99.52 ± 0.14 ***	99.13 ± 0.15 ***	98.61 ± 0.15 ***
MEAM 100	98.93 ± 0.17	100.04 ± 0.09 ***	99.82 ± 0.09 ***	99.60 ± 0.08 ***	99.40 ± 0.07 ***	99.02 ± 0.11 ***
MEAM 200	99.02 ± 0.15	100.47 ± 0.24 *	99.96 ± 0.18 ***	99.73 ± 0.19 ***	99.33 ± 0.14 ***	99.05 ± 0.20 ***
MEAM 400	98.97 ± 0.08	100.33 ± 0.14 **	99.96 ± 0.18 ***	99.54 ± 0.10 ***	99.10 ± 0.11 ***	98.82 ± 0.09 ***

* *p* < 0.05, ** *p* < 0.01, *** *p* < 0.001, statistically significant in comparison to control (Dunnett’s test).

**Table 6:** Molecular docking analysis of major bioactive compounds.

Compound Name	CID Number	Docking Score (kcal/mol)			
		1R4U	1A5H	2OYE	6COX
3,7,11,15-tetramethyl-2-hexadecen-1-ol	5366244	+0.007	−1.969	−2.941	−3.761
9-ecosyne	557019	+2.159	−0.30	−2.371	+0.318
Hexadecanoic acid	985	+1.829	−1.248	−1.663	+0.182
Gamma sitosterol	457807	−3.305	−3.379	−	-
Tridecanoic acid	12530	+1.374	+0.271	−0.822	−0.768
9-octadecenoic acid	965	-	−2.342	−2.107	−0.768
9,12-octadecadienoic acid	3931	-	−0.303	−2.229	−3.127
Canophyllal	12302400	−2.298	−4.228	-	−2.099
Canophyllol	7330581	−2.253	−3.973	-	−3.763
Friedelin	91472	−2.251	−4.241	-	-
Antidesmanol	44576122	−2.923	−4.181	-	−4.758
Carpusin	134369	**−5.113**	**−6.23**	**−7.613**	**−8.678**
Lupeolactone	21672669	−2.506	−3.802	-	-
Standard	-	Ascorbic Acid (−4.776)	Streptokinase (−6.173)	Diclofenac sodium (−7.545)	Diclofenac sodium (−6.917)

Bold texts indicate best docking scores.

**Table 7:** ADME/T (Absorption, distribution, metabolism, excretion, and toxicity) properties prediction of the bioactive compounds by SwissADME.

Compound Name	MW	HBA	HBD	Log P	MR	ROFV
3,7,11,15-tetramethyl-2-hexadecen-1-ol	296.53	1	1	6.22	98.94	1
9-ecosyne	278.52	0	0	7.51	96.42	1
Hexadecanoic acid	256.42	2	1	5.20	80.80	1
Gamma sitosterol	414.71	1	1	7.19	133.23	1
Tridecanoic acid	214.34	2	1	4.10	66.38	0
9-octadecenoic acid	282.46	2	1	5.71	89.94	1
9,12-octadecadienoic acid	280.45	2	1	5.45	89.46	1
Canophyllal	440.70	2	0	6.57	134.59	2
Canophyllol	442.72	2	1	6.57	135.56	2
Friedelin	426.72	1	0	7.45	134.39	2
Antidesmanol	442.72	2	1	6.51	135.56	2
Carpusin	302.28	6	3	1.50	77.34	0
Lupeolactone	438.69	2	0	6.96	133.15	2

MW, Molecular weight (acceptable range: ˂500 g/mol); HBA, Hydrogen bond acceptor (acceptable range: ≤10); HBD, Hydrogen bond donor (acceptable range: ≤5); Log P, High lipophilicity (acceptable range: <5); MR, Molar refractivity (acceptable range: between 40 and 130); ROFV, Rule of five violations (recommended range: 0–4).

**Table 8:** Toxicological properties of the selected compounds.

Compound Name	Ames Toxicity	Carcinogens	Acute Oral Toxicity	Rat Acute Toxicity
3,7,11,15-tetramethyl-2-hexadecen-1-ol	NAT	NC	III	1.6146
9-ecosyne	NAT	NC	III	1.6105
Hexadecanoic acid	NAT	NC	IV	1.3275
Gamma sitosterol	NAT	NC	I	2.6561
Tridecanoic acid	NAT	NC	IV	1.3275
9-octadecenoic acid	NAT	NC	IV	1.3991
9,12-octadecadienoic acid	NAT	NC	IV	1.3991
Canophyllal	NAT	NC	III	2.0526
Canophyllol	NAT	NC	III	1.7292
Friedelin	NAT	NC	III	1.9678
Antidesmanol	NAT	NC	III	2.2306
Carpusin	NAT	NC	III	2.7634
Lupeolactone	NAT	NC	III	2.4222

NAT, Non-Ames toxic; NC, Non-carcinogenic; C, Carcinogenic; Category I means LD_50_ ≤ 50 mg/kg; Category III means 500 mg/kg > LD_50_ < 5000 mg/kg; Category IV means LD_50_ > 5000 mg/kg.

## Supplementary Materials

The materials are available online.

## Abbreviations

MEAM, methanol extract of *Antidesma montanum*; TNF-alpha, tumor necrosis factor alpha; NF-κB, nuclear factor kappa-light-chain-enhancer of activated B cells; COX, Cyclooxygenase; ADME/T, absorption, distribution, metabolism, excretion, and toxicity; p.o., per oral; i.p., Intraperitoneal; BOD, biochemical oxygen demand; % MPE, Percentage of maximal possible effect; ROS, Reactive oxygen species; BSA, Bovine serum albumin; PBS, Phosphate-buffered saline; NSAID, Non-steroidal anti-inflammatory drug; DPPH, 1, 1, diphenyl-2-picrylhydrazyl; HRBC, Human red blood cell; GAE, Gallic acid equivalent; FCR, Folin–Ciocalteu reagent; QE, Quercetin; ANOVA, one-way analysis of variance; S.E.M., Standard error mean.
