# Supplementary material for: Insightful Valorization of the Biological Activities of Pani Heloch Leaves through Experimental and Computer-Aided Mechanisms
Source: Molecules. 2020 Nov 5;25(21):5153. doi: 10.3390/molecules25215153 (PMC7663931; doi:10.3390/molecules25215153)
Supplement: Supplementary file 1 [file molecules-25-05153-s001.pdf]

Supplementary Materials

Article

Insightful Valorization of the Biological Activities of Pani Heloch Leaves through Experimental and Computer-aided Mechanisms

Naureen Banu [1,†], Najmul Alam [1,†], Mohammad Nazmul Islam [1,†,*], Sanjida Islam [1], Shahenur Alam Sakib [1,2], Nujhat Binte Hanif [1], Md. Riad Chowdhury [1], Abu Montakim Tareq [1], Kamrul Hasan Chowdhury [1], Shamima Jahan [1], Afrina Azad [3], Talha Bin Emran [4,*] and Jesus Simal-Gandara [5,*]

[1] Department of Pharmacy, International Islamic University Chittagong, Chittagong 4318, Bangladesh; naureen2021@gmail.com (N.B.); nazmul9alam@gmail.com (N.A.); sanjida.ru@outlook.com (S.I.); sakibhasaniiuc@gmail.com (S.A.S.); nujhataanchol@gmail.com (N.B.H.); riadchy01@gmail.com (M.R.C.); montakim0.abu@gmail.com (A.M.T.); kamrulhasan73132@gmail.com (K.H.C.); shamimaj058@gmail.com (S.J.)

[2] Department of Theoretical and Computational Chemistry, University of Dhaka, Dhaka 1000, Bangladesh

[3] Bangladesh Council of Scientific and Industrial Research (BCSIR), Dr. Qudrat-i-Khuda Road, Dhanmondi, Dhaka 1205, Bangladesh; afrina.ru.pharm@gmail.com

[4] Department of Pharmacy, BGC Trust University Bangladesh, Chittagong 4381, Bangladesh

[5] Nutrition and Bromatology Group, Department of Analytical and Food Chemistry, Faculty of Food Science and Technology, University of Vigo—Ourense Campus, E32004 Ourense, Spain

* Correspondence: sayeadiiuc@gmail.com (M.N.I.); talhabmb@bgctub.ac.bd (T.B.E.); jsimal@uvigo.es (J.S.-G.); Tel.: +88-01674613722 (M.N.I.); +88-01819-942214 (T.B.E.); +34-988-387000 (J.S.G.)

† These authors contributed equally to this work.

Received: 11 October 2020; Accepted: 03 November 2020; Published: date

1. Qualitative Phytochemical Screening

The qualitative phytochemical analysis of the *MEAM* was carried out by the standard methodology for testing the alkaloid, flavonoids, proteins, cholesterols, resins, phenols, terpenoids, steroids emodines, tannins, and glycosides [1-3].

1.1. Test for Alkaloids

Two milliliters of extract solution was added with the 2-3 drops of Mayer's reagent, whereas the white precipitates considered as the presence of alkaloids.

1.2. Test for Flavonoid

One milliliter of extract solution was added with the few mL of lead acetate (10%) in a test tube, whereas the yellow precipitates indicated the presence of flavonoids.

1.3. Test for Proteins

Two milliliter of extract solution was added with the two mL of water and few drops of conc. HNO_3 in a test tube, whereas the yellow color indicated the presence of proteins.

1.4. Test for Cholesterols

Two milliliter of extract solution was added with the two mL of $CHCl_3$ and ten drops of acetic anhydrite and 2-3 drops conc. H_2SO_4 in a test tube, whereas the red-rose color indicated the presence of cholesterols.

1.5. Test for Resins

One milliliter of extract solution was added with the few mL of $C_4H_6O_3$ and 1 mL of conc. H_2SO_4 in a test tube, whereas the conversation of orange to yellow color indicated the presence of resins.

1.6. Test for Phenols

Five milliliter of extract solution was added with the 3 mL of lead acetate (10%) in a test tube and mixed very gently, whereas the white precipitates indicated the presence of phenols.

1.7. Test for Terpenoids

Three milliliter of extract solution was added with the 1 mL of chloroform and 2 mL of conc. H_2SO_4 in a test tube, whereas the reddish brown color indicated the presence of terpenoids.

1.8. Test for Steroids

Two milliliter of extract with 2 ml of chloroform and 2 ml of concentrated H_2SO_4 are added, the appearance of red color and yellowish green fluorescence indicates the presence of steroids.

1.9. Test for Emodines

To 5ml of extract, 2ml of NH3OH and 3ml of benzene are added. The production of red color indicates the presence of emodins.

1.10. Test for Tannins

Five milliliters of extract solution was added with the few drops of ferric chloride solution (5%), whereas the dark green color considered as the presence of tannins.

1.11. Test for Glycosides

The Borntrager's methodology was followed, whereas 2 mL of extract solution was added with 3 mL of chloroform and shaken well. After shaking, the layer of chloroform was separated and added ammonia solution (10%), whereas the pink color indicates the presence of glycosides.

References

1. Auwal, M. S.; Saka, S.; Mairiga, I. A.; Sanda, K. A.; Shuaibu, A.; Ibrahim, A., Preliminary phytochemical and elemental analysis of aqueous and fractionated pod extracts of *Acacia nilotica* (Thorn mimosa). *Vet Res Forum* **2014,** *5,* (2), 95-100.
2. Evans, W. C., *Trease and Evans' Pharmacognosy E-book*. Elsevier Health Sciences: 2009.
3. Hossain, M. S.; Reza, A.; Rahaman, M. M.; Nasrin, M. S.; Rahat, M. R. U.; Islam, M. R.; Uddin, M. J.; Rahman, M. A., Evaluation of morning glory (*Jacquemontia tamnifolia* (L.) Griseb) leaves for antioxidant, antinociceptive, anticoagulant and cytotoxic activities. *J Basic Clin Physiol Pharmacol* **2018,** *29,* (3), 291-299.
